# Does the Dream of Home Ownership Rest Upon Biased Beliefs? A Test Based on Predicted and Realized Life Satisfaction

**DOI:** 10.1007/s10902-022-00571-w

**Published:** 2022-09-14

**Authors:** Reto Odermatt, Alois Stutzer

**Affiliations:** Faculty of Business and Economics, Center for Research in Economics and Well-Being (CREW), University of Basel, Peter Merian-Weg 6, 4002 Basel, Switzerland

**Keywords:** Beliefs, Home ownership, Housing tenure, Life goals, Life satisfaction, Projection bias, Subjective well-being, Intuitive theories of happiness, Utility prediction, D12, D83, D90, I31, R20

## Abstract

The belief that home ownership makes people happy is probably one of the most widespread intuitive theories of happiness. However, whether it is accurate is an open question. Based on individual panel data, we explore whether home buyers systematically overestimate the life satisfaction associated with moving to their privately owned property. To identify potential prediction errors, we compare people’s forecasts of their life satisfaction in 5 years’ time with their current realizations. We find that home buyers for whom the purchase of the home is a main reason for moving, on average, systematically overestimate the long-term satisfaction gain of living in their dwelling. The misprediction therein is driven by home buyers who follow extrinsically-oriented life goals, highlighting biased beliefs regarding own preferences as a relevant mechanism in the prediction errors.

## Introduction

Many people hold the ambition of acquiring a home. Home ownership is considered part of the American dream and over 90% of the US population between the ages of 18 and 44 aim to own a house at some point in the future (Belsky, 2013; Goodman & Mayer, 2018). Even in Europe, where the average home ownership rate is lower, the majority of people would prefer to live in a privately owned property (Bourassa & Hoesli, 2010). Following the dream of home ownership is a major life choice. Apart from entailing major financial commitment, the decision about whether to purchase a house or apartment is also difficult. It involves many trade-offs with significant long-term consequences that are difficult to revoke, with few opportunities to learn from experience. If beliefs about the imagined benefits were biased, this may result in sub-optimal investment decisions. However, whether home buyers, on average, correctly estimate the benefits of moving to a privately owned home and, thus, hold accurate beliefs regarding the utility consequences of home ownership, is an open question.

In this paper, we propose and undertake an empirical test of the accuracy of home buyer’s beliefs about the well-being consequences of home ownership. In standard rational-agent models it is assumed that people, on average, hold unbiased beliefs about the utility consequences of their actions. However, recent behavioral economic studies have questioned this assumption and often refer to the model of a projection bias with people holding systematically biased beliefs about their future preferences (Loewenstein et al., 2003). Important empirical insights from studies in the field of affective forecasting suggest that people have the tendency to overestimate the initial impact and duration of an emotional event [see, e.g., (Wilson & Gilbert, 2003; Loewenstein & Schkade, 1999)]. These studies are complemented by experimental and survey research on “utility misprediction” [see, e.g., (Frijters et al., 2009; Hsee et al., 2012; Odermatt & Stutzer, 2019; Odermatt et al., 2021)] as well as field studies [see, e.g., (Acland & Levy, 2015; Busse et al., 2015)].^1^

Based on the idea that people might misperceive the utility consequences of outcomes, we hypothesize that the general belief about the preferability of owning compared to renting reflects an overestimation of the positive consequences of home ownership. In fact, there are a whole range of reasons nurturing the dream of home ownership. People associate home ownership with greater control over their lives, the promise of building wealth, less insecurity of tenure, higher-quality housing, better communities and—not least—social status [see, e.g., (Andersen, 2011; Belsky, 2013; McCabe, 2018; Reid, 2014)]. Such beliefs regarding the benefits of home ownership are important determinants of pre-purchase preferences for owning (Drew, 2014). Cohen et al. (2009) even argue that subjective perception about the preferability of home ownership is a stronger predictor of home purchase behavior than socio-demographic and financial characteristics. The important role of individuals’ beliefs in the housing market is underscored by evidence on the effect of superstition and motives for conspicuous spending on the demand for housing (see, e.g., (He et al., 2020) in a study for Singapore). In sociology, the corresponding ideas of housing aspirations are therefore treated as culturally mediated and socially constructed [see, e.g., (Nethercote, 2019; Ronald, 2008)]. Accordingly, housing aspirations might not necessarily equate to the real net benefits of home ownership.

We empirically test the basic hypothesis by assessing prediction errors around the buyers’ relocation to their purchased house or apartment. We exploit large-scale long-run panel data of the German Socio-Economic Panel (SOEP). In this annual survey, participants are asked about their individual life satisfaction, as well as how satisfied they expect to be in 5 years’ time. This allows us to calculate a measure for individuals’ accuracy in their prediction of future well-being. Specifically, we apply a recently developed strategy (Odermatt & Stutzer, 2019). We capture prediction errors around the status change from tenant to owner by estimating, first, the impact of the status change on individuals’ actual satisfaction over time, and, second, the impact of the status change on people’s prediction of their satisfaction 5 years into the future. The impact on expected life satisfaction can then be compared with actual changes in life satisfaction later on, with the difference between the two reflecting the prediction error. Our control strategy allows us to statistically abstract from other sources of prediction errors, in particular individual-specific and age-specific effects, taking account of potential selection effects that are prevalent in the analysis of home ownership.

Our results reveal that moving as a result of property purchase is associated with higher life satisfaction. However, home buyers, on average, are overly optimistic about the positive long-term satisfaction gains. This finding arises when we consider the predictions both just before and just after people have moved from a rented to a privately owned property. The analysis provides evidence that is consistent with the idea that home ownership, on average, positively contributes to people’s life satisfaction, but that home buyers also seem to hold overly optimistic beliefs about the *extent* to which the dream of home ownership will make them happy. In particular, the observation of systematically inaccurate predictions prior to the move suggests the relevance of biased beliefs in the decision-making process. Moreover, the analyses show that relocation to a different dwelling is crucial for the understanding of the prediction errors around a home purchase. This likely reflects that the trade-offs and uncertainties involved in the house purchasing decision particularly emerge with a relocation. In a supplementary analysis, we consequently study whether the misprediction of future well-being in the context of becoming a home owner might simply reflect that these people relocate, irrespective of whether it is to a rented or an acquired property. Moving to a new environment might generally be related to overestimating the positive impact on one’s life. We indeed find that prediction errors also occur when people relocate to a rented home. However, the errors are substantially smaller compared to when people move to an acquired property and this acquisition serves as the main reason for moving. This suggests that our main findings reflect more than just the relocation to a new environment.

We further consider that people’s beliefs might deviate from the correct ones for different reasons. People might hold biased beliefs regarding the probabilities of possible decision outcomes, as well as regarding their preferences and the extent to which these might change. In order to additionally and more directly test whether the prediction errors result from an individual’s incomplete knowledge of his or her preferences, we consider the heterogeneity in people’s life goals as a proxy for different underlying beliefs about their own preferences. Specifically, we investigate whether relying on an extrinsic value orientation contributes to an overestimation of the positive consequences of home ownership. Based on findings in the literature (Sheldon et al., 2010), we conjecture that for home buyers with extrinsically-oriented life goals the prediction errors with respect to home ownership are more pronounced. Indeed, we find that home buyers who value things like income, success, and the ability to buy goods relatively highly commit significant errors, while the others do not.

We organize the remainder of the paper as follows. Section 2 presents the theoretical considerations along with a selection of related empirical findings, and derives the hypotheses. The data and empirical strategy are described in Sect. 3. The estimation results are presented in Sect. 4. Section 5 offers concluding remarks.

## The Role of (Biased) Beliefs in Choice and Well-Being

### Beliefs about Probabilities and Preferences

In standard expected utility theory, people pursue their goals optimally. Specifically, people behave optimally given their beliefs, and correct beliefs (or optimal learning) are assumed, i.e., beliefs and resulting choices that in expectation maximize their well-being. These beliefs might refer to the probabilities of possible decision outcomes (like the riskiness of some asset category) or to an individual’s current or future preferences. However, if people hold inaccurate beliefs, the materialization of their choices contributes less to the fulfillment of their life goals than what would have been possible with accurate ones.

Many studies provide evidence for a discrepancy between objective and subjective probabilities. Such a discrepancy reflects biased beliefs about the probability of states of the world or characteristics of goods. A general finding is that agents tend to overestimate the probability of positive outcomes and underestimate the probability of negative ones (Weinstein, 1980). This may be due to overoptimism [(van den Steen, 2004) or (Sharot, 2011) for a review], overconfidence [(Barber & Odean, 2001) or (Malmendier & Tate, 2005), and (Moore & Healy, 2008) for a review] or salience, if decision makers overweight salient states (e.g., (Bordalo et al., 2012)).

In contrast, there is much less systematic knowledge about inaccurate beliefs that people hold about their (specific) preferences, i.e., what they like.^2^ This comprises that people have to form beliefs in substantive areas of their life, such as how they would enjoy or derive satisfaction from some state of the world (like winning the lottery, having children, owning a house) or some activity (like opening up a bar, climbing Kilimanjaro). Differences in these beliefs are also a reflection of people’s values, such as their view on what goals should be pursued in life to satisfy needs. However, not every goal may lend itself equally well to the pursuit and achievement of high well-being.

In the following, we substantiate these ideas for the concrete case of the acquisition of residential property and the pursuit of material life goals. In Sect. 2.2, we reason how biased beliefs might lead to systematic prediction errors with regard to home ownership. And in Sect. 2.3, we discuss extrinsic value orientation (or extrinsic life goals) as a possible source of biased beliefs.

### Beliefs Regarding Home Ownership

Prospective and current home owners process information on a wide range of topics to form beliefs about the attractiveness of owning rather than renting. Various studies observe patterns consistent with the dream of home ownership. However, there are also studies documenting potential negative aspects of home ownership.

The attractiveness of home ownership is reflected in a positive relationship with social commitment (DiPasquale & Glaeser, 1999) or community interactions (Hoff & Sen, 2005), local political participation (Manturuk et al., 2009), the upbringing of children (Green & White, 1997), physical health (Pollack et al., 2010) or the satisfaction with housing [(Elsinga & Hoekstra, 2005; Diaz-Serrano, 2009), and (Stotz, 2019)]. However, there are also studies which emphasise the negative aspects of home ownership, such as greater immobility in the labour market or more investment risk due to a less diversified portfolio (see, e.g., (Blanchflower & Oswald, 2013); and (Dietz & Haurin, 2003) for a review of positive and negative micro-level consequences). Tumen and Zeydanli (2014) even find a negative relationship between the transition from non-ownership to ownership on self-reported job satisfaction scores, particularly in the long run due to reduced mobility.

An appropriate weighing up of the advantages and disadvantages of home ownership against each other is a challenge when assessing its consequences on individual welfare. Many studies use self-reported life satisfaction as a proxy measure for individual welfare to gauge the overall effects of home ownership on an individual level. In line with the belief that home ownership makes people happy, studies typically find a positive correlation between home ownership and subjective well-being (see, e.g., (Rossi & Weber, 1996) for the United States, (Stillman & Liang, 2010) for Australia; (Ruprah, 2010) for Latin America, (Hu, 2013) for urban China, (Zumbro, 2014), and (Clark & Diaz Serrano, 2020) for Germany, or (Seiler-Zimmermann & Wanzenried, 2019) for Switzerland).

Studies that directly refer to beliefs regarding home ownership mostly relate to beliefs about the financial consequences of a house purchase. Glaeser et al. (2008), for example, claim that general findings regarding overoptimism about future prices can be applied to housing economics. They further argue that any biases have major consequences, because in the housing market transaction costs are higher and short-selling is more difficult than in almost any other asset market. Belsky (2013) refers to a survey by Case and Shiller (2003) which shows that expectations about the future growth in house prices are generally biased towards the present market environment, a potential driver of housing bubbles. Given this rationale, people tend to underestimate the costs of home ownership, revealing flawed reasoning in their judgment of the financial superiority of ownership over tenancy (Ben-Shahar, 2007). In addition, Bucks and Pence (2008) find that borrowers with adjustable-rate mortgages are likely to underestimate or not to understand the extent of possible rate increases from year to year or over the life of their loan, implying that they underestimate the risk of higher future interest rates.

Misbeliefs about the favourability of home ownership might also occur when individuals have incomplete knowledge of their preferences.^3^ A study by Dunn et al. (2003) investigates prediction errors of undergraduate students regarding their predicted happiness about the potential dormitories that they could be assigned to. They find that the students placed far greater weight on physical features than on social features when predicting their future happiness, although social features turned out to be more relevant for their happiness later on. Mispredicted adaptation might also play a role in the housing market. Hoelzl et al. (2009) conduct a survey of 117 home owners at different stages of the loan process. They find that people erroneously expect that their negative emotional experience of the loan burden will decrease over time. This finding suggests that home owners hold an incorrect belief about their capacity to adapt to a burdensome financial situation, resulting in an overestimation of the long-term satisfaction benefits.

In sum, while there are many benefits to owning rather than renting, the net advantages might be misperceived. In fact, there is evidence supporting the behavioral economic conjecture that people hold systematically biased beliefs regarding the long-term benefits of home ownership: People are generally too optimistic about future circumstances in the housing market, tend to apply inappropriate weights to different attributes of housing, and underestimate the long-term negative impact of carrying a financial burden. Based on these findings, we postulate the following hypothesis:

#### Hypothesis 1

Home buyers overestimate the long-run life satisfaction gains derived from moving from a rented home to a privately owned property.

Please note, however, that theoretically the overall prediction error could go in both directions if there is a systematic change in circumstances that requires people to adjust their beliefs about the world. For example, if the circumstances on the housing market were to systematically change during our observation period and new home owners would hold systematically too pessimistic (or not sufficiently optimistic) beliefs about the increase in house prices, the observed prediction error would, on average, be negative. As we can only control for time fixed effects that are independent of the tenure status, such a scenario would also be possible in our setting.

### Extrinsic Value Systems as a Source of Biased Beliefs

People hold different intuitive theories about the sources of utility, such as beliefs about what goals should be pursued in life to satisfy needs. As discussed, these beliefs can be erroneous in the sense that the expected utility does not materialise even if the specific goal is achieved. Traditional economic research takes goals as given and does not ask for their specific content, as they are reflected in people’s preferences. In order to study differences in people’s beliefs about their preferences, we thus rely on the insights of a rich related literature in the social sciences that tries to understand heterogeneity in people’s goals, often referred to as differences in value systems. Such differences in value systems, in turn, allow us to approximate differences in the beliefs people hold about their preferences. One prominent distinction is between an extrinsic and an intrinsic value system (see, e.g., (Tatzel, 2002) for a discussion in the field of economics). With an extrinsic system, financial success and material possession are pursued, while a non-materialistic or intrinsically oriented system promotes the satisfaction of personal, intrinsic values, such as social relationships, family, and experiences. However, these value or belief systems need not all be equally good at representing the extent to which certain goods are beneficial to individual welfare.

There are arguments in the economic literature which claim that extrinsic value systems generate false motivational goals. They lay too much weight on material goods and induce individuals to undervalue goods that provide non-material benefits [see (Scitovsky, 1976) or (Frank, 1999)]. Indeed, many studies report that people who follow materialistic or extrinsic life goals report lower life satisfaction than those who follow intrinsic life goals [e.g., (Sirgy, 1998; Kasser, 2002) or (Sheldon et al., 2004)]. Sheldon et al. (2010) argue that this difference can be explained partly by suboptimal behavior, because extrinsically oriented people are prone to overestimating the emotional benefits of consuming materialistic goods. Consequently, these people potentially misallocate their time, effort and money, and in turn derive a lower level of individual welfare.^4^

Extrinsic value systems might be relevant to perceptions of home ownership from various perspectives. Housing in general can be seen as a multi-attribute good that satisfies extrinsic as well as intrinsic needs. Regarding the former, Elsinga and Hoekstra (2005) and Ronald (2008) provide reviews of theories about the meaning of home ownership that highlight its extrinsic dimension. In particular, home ownership is related to perceived higher social status, and the purchase of property is regarded as a significant “achievement” [see, e.g., (Rohe et al., 2002) or (Reid, 2014)]. Additionally, the preference for home ownership can partly be explained by a “possessive instinct” that people have and their desire to mark out their own territory (Saunders, 1990). Moreover, Bellet (2019) shows evidence for positional externalities in the housing market, emphasizing the extrinsic dimension of home ownership. Regarding intrinsic needs, people identify home ownership with better communities, more control over living space, and living arrangements that are beneficial for one’s family (Belsky, 2013). The relevance of the domains might be differently weighted in the valuation of home ownership, depending on what life goals people pursue. Extrinsically oriented home buyers might put more weight on the extrinsic aspects of a house purchase than intrinsically oriented home buyers, and vice versa. If we combine the different arguments, we come to the conjecture that people with extrinsic life goals are particularly prone to holding biased beliefs about the benefits of materialistic goods. Accordingly, we formulate the following second hypothesis:

#### Hypothesis 2

Home buyers with extrinsically oriented life goals overestimate the long-run life satisfaction gains derived from moving from a rented home to a privately owned property to a greater extent than home buyers with intrinsically oriented life goals.

## Data and Methodology

### Data and Sample Selection

We base the empirical analysis on individual-level panel data from the German Socio-Economic Panel (SOEP) (Wagner et al., 2007). This representative survey of the German population has been conducted annually since 1984 and contains a wide range of questions regarding socio-economic status and demographic characteristics. Importantly, every year respondents report their subjective well-being by answering the question: “How satisfied are you with your life, all things considered?”. In many of the years since 1991, people were then asked the question: “And how do you think you will feel in five years?”. People answer both questions according to an eleven-point satisfaction scale from 0 meaning “completely dissatisfied” to 10 meaning “completely satisfied”. This provides the information for identifying our key dependent variables in the subsequent empirical analysis. Specifically, we use data from 1991 to 2013 and exclude the years in which the survey did not include the item about satisfaction with life in 5 years, which was in 2005–2007 and 2010.^5^

In addition to querying people’s predicted and actual satisfaction with life, it is necessary to identify transitions to home ownership. To do so, we exploit the information in the SOEP regarding the tenure status of respondents. We consider the status change from tenant to owner across two surveys as indicating a person’s transition from being a tenant to becoming home owner. We only consider the first time that the status change in question occurs for an individual within the sample period and exclude respondents who switched to home ownership before entering the survey (left-censored spells). We further require a full record of the tenure status without missing years, which ensures that we have observed all status changes. We only consider those home owners whose status change occurred in the period between 1991 and 2009.

We focus our analysis on purchase decisions that require relocation to a different dwelling. These cases involve substantial uncertainty and require a comprehensive formation of beliefs.^6^ In order to differentiate between non-movers and movers, we make use of information about people’s relocation behavior provided in the SOEP. Respondents are asked whether and when they moved since they were interviewed the previous time. We restrict the sample in our main analysis to those respondents for whom the moving date is available. The moving date allows us, in combination with the interview date, to calculate the distance to the move in months and years. Relying on the moving date (rather than the purchase date) ensures that the time structure is precisely linked to the experience of the new housing situation.

In a further sample restriction, we address the challenge to the analysis that the point of time at which people purchase a house is endogenous to the experience of circumstances that potentially relate to subjective well-being. The reasons why people decide to purchase their own home are manifold, be it a change in the family situation or a new job. The status change to ownership might then be more of a by-product of another important (potentially omitted) life decision. In order to capture status changes that are specifically related to the decision to purchase a dwelling, the analysis therefore needs to exclude observations primarily driven by other factors. We do this based on a survey item that queries the reason for the relocation. The respondents have the option to indicate that they moved because they bought a house or an apartment. Or, they can state a different cause for their relocation, such as being noticed, an inheritance, job- or family-related reasons (e.g., marriage, divorce, children) or reasons related to the characteristics of the dwelling (e.g., size, cost, location). We concentrate on those home owners who indicate the purchase of property as a main reason for moving, without mentioning any of the other possible reasons.

These restrictions leave us with 839 individuals whom we classify as changing status from tenant to owner, providing us with 8811 person-year observations around transitions to home ownership. Regarding age, we limit the sample to respondents who are 18 years of age or older and younger than 90 years of age. In total, we use a sample of 126,276 observations, including both people who acquired property and tenants. Details of the empirical strategy are discussed in the following Sect. 3.2. Table 3 in the Appendix presents the descriptive statistics. It reports the mean values and standard deviations of variables involved in the empirical analysis. They are shown for the sample used in our preferred specification. The characteristics of home buyers and tenants are shown separately.^7^

### Empirical Strategy

To identify potential prediction errors when people become home owners, we apply the strategy proposed by Odermatt and Stutzer (2019) for identifying prediction errors around life events. In a regression with individual fixed effects, we include separate indicators for the years around an individual’s status change to capture movements in current and predicted subjective well-being. Accordingly, we compare the changes in predicted life satisfaction with the actual changes in life satisfaction 5 years later.

Specifically, we estimate two models of the following form:

1 $$\begin{aligned}&\begin{aligned} PS_{it} = \alpha _{i} + \sum _{j=-4}^6 \theta _{j}{} Owner ^{j}_{it} + \beta {'}{} {\textbf {X}}_{it} + \varepsilon _{it} \end{aligned} \end{aligned}$$

2 $$\begin{aligned}&\begin{aligned} LS_{it} = \alpha _{i} + \sum _{j=-4}^6 \theta _{j}{} Owner ^{j}_{it} + \beta {'}{} {\textbf {X}}_{it} + \varepsilon _{it} \end{aligned} \end{aligned}$$

Predicted and current life satisfaction serve as dependent variables. *PS*$_{it}$ stands for the predicted life satisfaction of individual *i* at time *t*, and *LS*$_{it}$ stands for the realized actual life satisfaction of individual *i* at time *t*. **X**$_{it}$ is a vector of individual controls indicated in Table 3. The main explanatory variables are given by the series of dummy variables *Owner*$^{j}_{it}$, indicating the number of periods *j* before and after the status change to ownership. The first dummy captures observations 3 to 4 years before the status change. The last dummy captures the reports of home buyers six or more years after they experienced the status change. This means that the reference category consists of all the years up until 4 years preceding the status change. The inclusion of individual fixed-effects ${\upalpha }_{i}$ results in within-individual estimates. This controls for any time-invariant individual characteristics, and implies that the partial correlations are only based on variation within the same person over time. It first rules out that individual-specific optimism or pessimism drives the differences between predicted and experienced life satisfaction, in particular tenants’ and prospective home owners’ general tendency to over- or underestimate future satisfaction with life. Second, it takes account of potential selection that is due to home owners sharing underlying characteristics associated with, for example, a higher satisfaction with life. In addition, the vector of control variables includes age-specific fixed effects capturing changes in our dependent variables which are common for a particular age group, such as aspects of maturity, or the tendency of individuals to overestimate their life satisfaction in the first half of their life span. Time-fixed effects are further included to control for systematic changes over time that are common to all individuals, such as changes in the federal subsidy programs [see, e.g., (Stotz, 2019)]. Lastly, region-fixed effects control for regional characteristics that might be correlated with our variables of interest.

Our estimation sample includes both people who acquired and who did not acquire property. The latter, i.e., those remaining tenants throughout the observation period, experienced the counterfactual situation to owning. Including both groups allows us to better separate the impact of the status change from the systematic fluctuations in satisfaction measures over time, which are captured by the year-specific time dummies. Moreover, the coefficients of our control variables are more precisely estimated, which in turn increases the efficiency of the estimation of our key coefficients.

The empirical measures to test Hypothesis 1 are determined by the difference between the coefficient ${\theta _{j}}$ of model (1) and ${\theta }_{j+5}$ of model (2), with $H_{0}$: ${\theta }_{j}^{PS}-$ ${\theta }_{j+5}^{LS}=0$.^8^ For example, the difference ${\theta }_{0}^{PS}-$ ${\theta }_{5}^{LS}$ reflects the average individual prediction error in the first period after moving from a rented to a privately owned property. A significant positive difference (rejection of $H_{0}$) provides support for the hypothesis that home buyers overestimate the long-run life satisfaction gains of the status change to home owner, conditional on the average individual-specific errors 4 years or more before ownership. Estimating two separate profiles has the advantage that, first, it shows the impact of the event separately on home buyer’s predicted life satisfaction as well as on their actual life satisfaction. Second, compared to an estimation strategy that uses prediction errors directly as dependent variable, demands regarding data are smaller, mainly because we do not need more years of observation before the event occurs in order to control for errors due to the misprediction of the house purchase itself and the timing of it in the reference period.

We focus on the predictions made just before and after the status change. In order to approximate the beliefs that were probably relevant for the decision to buy, we look at home buyer’s predictions shortly *before* they are due to move to their new dwelling. Specifically, we look at the predictions made within the last four months before the status change. Within this period, they likely know about the properties of the new dwelling and are aware that their future life will involve home ownership.^9^ Given the knowledge and salience of the status change, it is likely to be incorporated into home buyer’s predictions about their life satisfaction in 5 years’ time. Thus home buyer’s expectations about the well-being consequences of their decision are revealed before experiencing them. In other words, we empirically test the accuracy of their beliefs about the total benefits of home ownership.

By studying the predictions that home buyers make shortly *after* moving to their purchased dwelling, we are able to investigate whether they correctly anticipated the degree of their adaptation to the new status as home owners once they were established in their new living circumstances for some time. In particular, we look at the predictions made within four and twelve months after the status change.

## Empirical Evidence for Biased Beliefs

### Changes in Expected and Actual Life Satisfaction of Home Buyers

**Fig. 1:**
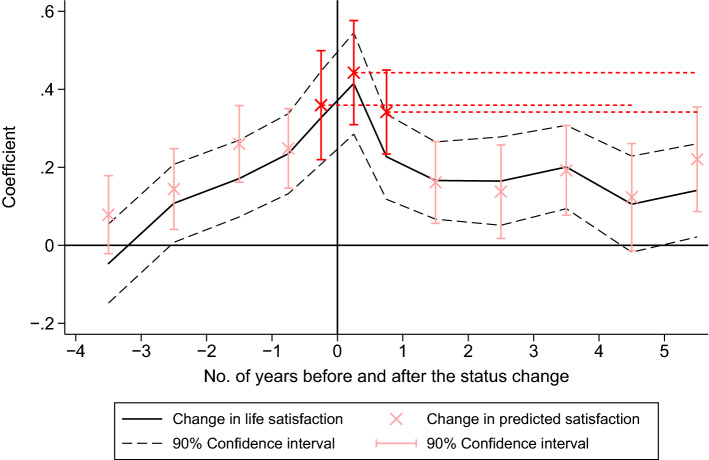
Estimated patterns in actual and predicted life satisfaction around the status change to home owner. *Note*: This figure is based on the estimated coefficients in Table 1. The black solid line shows the coefficients of specification II and the red x-marks indicate the coefficients of specification I. The red dashed lines are auxiliary lines that project the predictions to the corresponding periods 5 years later. The prediction errors for the three comparisons are reflected in the differences between the red dashed lines and the black solid line (capturing the effect on actual satisfaction) in the period at the end of the red dashed line *Source*: SOEP

In this section, we present the results for the estimations of changes in expected and actual life satisfaction around the transition to home ownership. Table 1 presents the results for the models outlined in equations (1) and (2), and Fig. 1 presents the estimated coefficients graphically for ease of interpretation.^10^ The column labelled PS presents the estimate with predicted life satisfaction as the dependent variable. The column labelled LS shows the estimate with current life satisfaction as the dependent variable. The results are for our preferred sample specification (also referred to in the descriptive statistics), which is based on the restrictions outlined in Sect. 3. It focuses on home buyers for whom the purchase of the home is the reason for moving. The coefficients in the second column show the changes in individual life satisfaction in the years around relocation due to the acquisition of a house or an apartment. The estimates indicate a significant improvement in home buyer’s life satisfaction. Compared to their baseline level of subjective well-being four or more years prior to home ownership (i.e., the reference period), reported life satisfaction as indicated on the eleven-point satisfaction scale is 0.415 points higher in the first four months after the purchase. This indicates a substantial short-term satisfaction benefit that is even higher, for example, than the satisfaction increase when people get married [see e.g., (Stutzer & Frey, 2006)].

**Table 1:** Regression of predicted (PS) and actual life satisfaction (LS) around home ownership: Prediction errors calculated for the predictions made in the months before and after the status change *Source*: SOEP

	PS	LS
	I	II
*Before ownership*		
4–3 years hence	0.079	− 0.047
	(0.06)	(0.06)
3–2 years hence	0.144**	0.108*
	(0.06)	(0.06)
2–1 years hence	0.260***	0.171***
	(0.06)	(0.06)
12–5 months hence	0.248***	0.235***
	(0.06)	(0.06)
4–0 months hence	0.359***	0.327***
	(0.08)	(0.07)
*After ownership*		
0–4 months	0.443***	0.415***
	(0.08)	(0.08)
5–12 months	0.342***	0.227***
	(0.07)	(0.07)
1–2 years	0.161**	0.166***
	(0.06)	(0.06)
2–3 years	0.137*	0.165**
	(0.07)	(0.07)
3–4 years	0.192***	0.201***
	(0.07)	(0.06)
4–5 years	0.123	0.105
	(0.08)	(0.07)
5–6 years	0.220***	0.141*
	(0.08)	(0.07)
6 or more years	0.306***	0.179***
	(0.07)	(0.07)
*Differences*		
PS(4–0 months)–LS(4–5 years)	0.254***	
	(0.102)	
PS(0–4 months)–LS(5–6 years)	0.302***	
	(0.098)	
PS(5–12 months)–LS(5–6years)	0.201***	
	(0.084)	
Individual controls	Yes	Yes
Age fixed effects (FE)	Yes	Yes
Time and region FE	Yes	Yes
Individual FE	Yes	Yes
No. of observations	126,276	126,276
No. of individuals	25,081	25,081
No. of home buyers	839	839
R$^2$	0.04	0.04

Standard errors in parentheses. Significance levels: *$.05<p<.1$, **$.01<p<.05$, ***$p<.01$. Significance levels of the prediction errors derived from a z-test

Two further aspects stand out with regard to the effect of home ownership on life satisfaction. First, the long-term impact on satisfaction 5 to 6 years after purchase is indicated as being 0.141 points. This suggests that there is substantial, albeit not complete, adaptation to the initial positive effect. Second, given that the responses record significant satisfaction increases in the years and particularly in the month prior to the actual status change, there seem to be substantial anticipation effects. However, while we control for many important life circumstances, this pattern might also reflect beneficial living conditions not controlled for which are potentially correlated with the imminent purchase.^11^

A similar profile is estimated for home buyer’s predicted life satisfaction in 5 years’ time. In the first four months after the status change, our statistical analysis shows that home buyers expect their long-term satisfaction levels to be 0.443 points above the baseline predictions that they made four or more years earlier. In the eight months thereafter, the difference is still 0.342 points. Interestingly, the relocation seems to have a stronger impact on predicted life satisfaction than it does on actual life satisfaction, suggesting that home buyers, on average, expect their life satisfaction to increase even further over subsequent years. Furthermore, home buyers expect a higher level of life satisfaction in 5 years’ time already in the months and even years before they actually move to their purchased dwelling.

### Prediction Errors Before and After Moving to the Acquired Property

As illustrated in Fig. 1, the increases in home buyer’s predicted satisfaction in the months around the status change are larger than the actual long-term changes in life satisfaction, indicating sizeable prediction errors. Based on the coefficients in Table 1, we can calculate the exact size of the average error made in the months before the status change. This is done by looking at the difference between the impact on predicted life satisfaction shortly before the event (the coefficients for *4–0 months hence* in specification I) and the impact on actual satisfaction 5 years later (i.e., the coefficient for *4–5 years* in specification II). The potential prediction error in the months after the status change is the difference that results when the actual impact (the coefficient *5–6 years* in specification II) is subtracted from the predicted impact (the coefficients *0–4 months* or *5–12 months* in specification I). In all three cases, the estimates indicate statistically significant differences between predicted and realized life satisfaction (see Table 1).

**Fig. 2:**
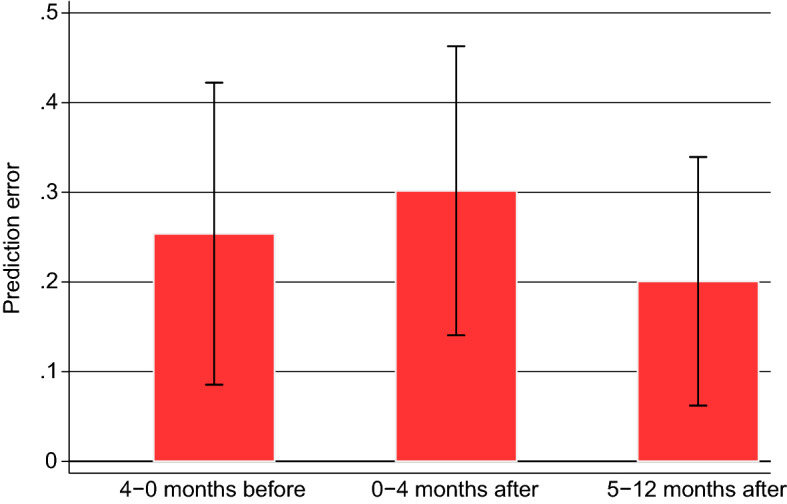
Graphical representation of the prediction errors before and after the status change to home owner. *Note*: 90% confidence intervals are indicated. *Source*: SOEP

Figure 2 provides a summary of the estimated sizes and patterns in the prediction errors. In line with Hypothesis 1, it reveals that home buyers, on average, are overly optimistic about the long-term consequences of home ownership. This holds before and also after they have moved into their privately owned dwelling. The calculated prediction errors are, moreover, not only statistically significant but also sizeable. Before moving, they amount, on average, to 0.254 points. This is more than a third of the difference in life satisfaction when the same people are observed to be unemployed rather than employed (see Table 4). In the first four months after moving, the prediction error seems to be even larger, i.e., 0.302 points, although it cannot be rejected that it is of equal magnitude to just before moving. Even in the five to twelve months after the status change a prediction error of 0.201 is estimated. This suggests an overestimation of the long-term benefits of home ownership that is not due to mistaken beliefs about the immediate experience.^12^

### Sensitivity Analyses

**Fig. 3:**
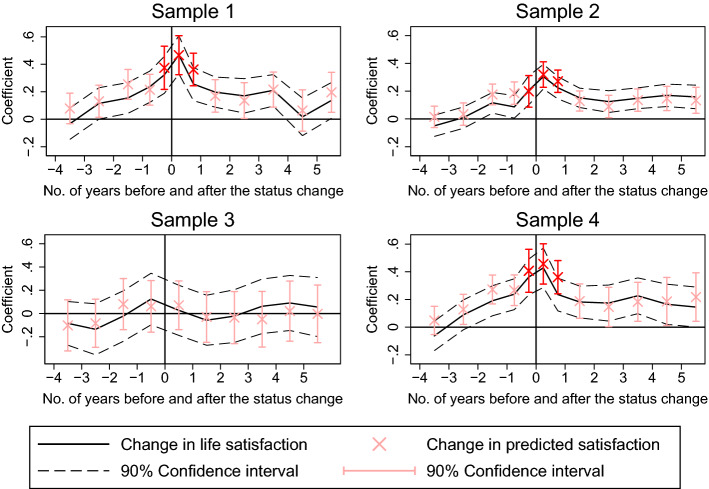
Estimated patterns in actual and predicted life satisfaction around the status change to home owner for different samples. *Note*: These graphs are based on the estimated coefficients of samples 1–4, specifications I–VIII in Table 5. For a description of the reading of the graphs, see Fig. 1. Sample 1 consists only of home owners who acquired a house (and not an apartment). Sample 2 comprises all individuals for whom we observe a status change from tenant to owner. Sample 3 refers to people who become owners without changing dwellings in the previous, the current, or the upcoming year. In Sample 4, the main specifications are estimated using entropy balancing to make tenants more comparable to home buyers *Source*: SOEP

To assess the sensitivity of the estimated prediction errors with regard to the selection of the sample, we estimate the profiles introduced above for four additional samples. Figure 3 presents the graphical representation of all the estimated profiles, while the regression results are presented in Table 5.

First, we re-estimate the profiles with a sample that includes only house owners (i.e., not apartment owners). Acquiring and moving into a house instead of an apartment reflects more directly the dream of home ownership and likely involves a greater change in circumstances. We thus expect larger prediction errors for this sample. This expectation seems confirmed as reported in specifications I and II in Table 5. When the 228 individuals who acquired an apartment instead of a house are excluded, the calculated prediction errors are slightly more pronounced. In particular, the error for the four months before the status change now amounts to 0.375 points. The estimates further suggest that the effect on life satisfaction is slightly higher the year after the purchase compared to specification II in Table 1, although a similar long-term benefit is estimated.

Second, sample 2 comprises all the individuals for whom we observe a status change from tenant to owner, irrespective of their relocation behavior and the reasons for purchasing a home. This all-encompassing sample definition probably involves cases for which the status change to home owner was a side effect of some other decisions. Accordingly, we expect the prediction errors to be attenuated. And indeed, when estimated for this broad sample of 2136 new home owners, they are still positive but smaller in magnitude compared to the errors in our main specification and only statistically significantly different from zero for the predictions after the status change. The smaller prediction errors arise due to slightly smaller changes in the predictions in the months around the transition to home ownership as well as longer-lasting life satisfaction gains after the transition. Finding smaller prediction errors when we do not require respondents to state the purchase of property as a main reason for moving is consistent with the thesis that the dream of home ownership is one of the main drivers of the prediction error. In other words, if other reasons drive the change in tenure, overly optimistic predictions are not as prevalent.^13^

Third, in sample 3, we estimate the profiles for all those home owners who are not observed to have moved one survey prior to or within two surveys subsequent to becoming home owners. This sampling approximately captures the group of individuals who purchased the dwellings they had already been living in. The corresponding prediction error could thus reflect the prediction error about ownership itself. However, in these cases, many of the trade-offs and uncertainties usually involved in the house purchasing decision do not emerge. Consequently, we expect less distinct prediction errors if there are any at all in this sample. Specifications V and VI do not reveal a significant impact of the status change on life satisfaction per se, and accordingly, no prediction errors are present.^14^ The relocation to a different dwelling is thus crucial for the understanding of the positive effects of home ownership and the prediction errors.

Fourth, in a last robustness exercise (sample 4), we use a matching strategy to deal with the situation that some of the characteristics of home buyers and tenants differ systematically (as indicated in Table 3 in the Appendix). Specifically, we re-weight the sample of tenants using entropy balancing (Hainmueller & Xu, 2013) so that their average characteristics match those of the sample of home owners in the period before they acquire property.^15^ The estimation results in Specifications VII and VIII and the corresponding prediction errors are not statistically significantly different from the ones presented as main results.

In sum, the results of the sensitivity analyses suggest that people who move to become the owners of a house (but also of an apartment) on average overestimate the positive long-term benefits of ownership and that this effect is not due to different socio-demographic characteristics of the new owners compared to tenants.

### Comparison with Rental Relocation

We evaluate people’s prediction for the experience of moving into one’s own property. This life decision usually involves at least two aspects, i.e., to become a home owner and to move. These changes in circumstances are difficult to unbundle into additive components that could then be separately assessed in terms of their contribution to any misprediction. However, a first attempt was presented in the previous section where we focus on people who purchased the dwellings they had already been living in (based on a small sample though). Here we present a second attempt by comparing the results for moving into one’s own property with the relocation that does not involve a property change, i.e., changing between rented apartments or houses. If for rental relocation the same prediction error were to be observed, it would be difficult to maintain that the aspect of acquiring property is a specific source for potential misprediction.

We thus undertake the same analyses as presented for our main sample in Sect. 4.1 for the sample of renters who relocate. The regression output as well as the graphical representation of the coefficients of interest are presented in the Appendix (see Table 6 and Fig. 5). We observe that renters who make predictions in the first year after the move are also overly optimistic regarding their future life satisfaction. However, they are not before the move. Moreover, the prediction errors after the move are substantially smaller in size amounting to 0.10 to 0.15 units rather than the 0.20 to 0.30 for new home owners in our main analysis (where we concentrate on those home owners who indicate the purchase of property as a main reason for moving, which most likely reflect those who pursue the dream of home ownership). However, if we relax this sample restriction and compare the size of the prediction errors to all home buyers who move (sample 2 in Sect. 3), we do not observe a significant difference. We conclude that if home ownership is indicated as a main reason for moving, the estimated prediction errors of home buyers reflect more than relocating to a new (and only partly known) environment.

### Heterogeneity in Beliefs: Extrinsic Value Systems as a Source of Prediction Errors

In this section, we investigate Hypothesis 2 that the prediction errors are larger for home buyers with extrinsically oriented life goals than for home buyers with intrinsically oriented ones. Considering the heterogeneity in people’s life goals as a proxy for people’s underlying beliefs about their preferences allows us to directly test whether the prediction errors result from an individual’s incomplete knowledge of his or her preferences. Accordingly, we assess whether the difference between predicted and realized satisfaction with life is systematically greater for the former than for the latter. To implement this test, we categorise the individuals with regard to their value orientation. In this, we make use of a series of questions included in the SOEP that investigate the importance individuals attribute to certain areas of life. The questions are based on a classification of life goals, initially developed by Kluckhohn and Strodtbeck (1961), that aims at measuring three domains: materialism (as well as achievement and success), family life, and altruism (for a discussion of the development and the reliability of the measures, see (Headey, 2008)). In the surveys, respondents are asked to rate the importance they attach to certain life areas on a 1 to 4 scale ranging from “not important” to “very important”.^16^

Using principal component analysis, Headey (2008) and Headey et al. (2010) classify the importance of being able to buy things, being able to achieve one’s potential, and being successful in one’s job relating to the success domain. We adopt this categorisation and take these items as indicators of extrinsic value orientation. We further add the item on the importance of income to this group, an item that was not used in their analyses. For the indication of intrinsic value orientation, we use items relating to the domains of family life and altruism, namely the importance of family, friends, being there for others, and being politically/socially involved. Table 7 provides an overview of the items and years that are used.

To differentiate between people with a predominantly extrinsic versus intrinsic value orientation, we focus on the relative importance that people attach to one or the other type of values. Specifically, for every individual in the sample, we use the earliest observation per item and calculate the mean across all the intrinsic and all the extrinsic items. As we are interested in the value orientation expressed in the period around the decision and purchase process, we only include those home owners whose importance measures are recorded up until the first year after the purchase. The ratio of the mean values of the extrinsic and intrinsic items therefore expresses the importance of extrinsic values relative to the intrinsic ones. The median of this measure for the sample of home owners serves as the threshold value to form two groups: We classify all individuals with a value higher or equal to the median as extrinsically oriented, and all those below the median value as intrinsically oriented.

Table 8 presents the descriptive statistics for the two samples of extrinsically and intrinsically oriented individuals. When we compare the mean values of the socio-demographic characteristics, we see that extrinsically oriented people tend to have lower actual and predicted satisfaction with life than intrinsically oriented people. In addition, the extrinsic sample comprises relatively more males, people of younger age, and more unmarried individuals, on average. Also, more individuals who are currently working and fewer pensioners are classified as extrinsically oriented. This is not surprising, as the importance of success in a job is included in the measure for the extrinsic value orientation. However, the average household income after tax is rather similar in the two samples, despite the inclusion of the item on the importance of income in the extrinsic domain.

**Table 2:** Regression of predicted (PS) and actual life satisfaction (LS) around the status change to home owner for the samples of extrinsically and intrinsically oriented people *Source*: SOEP

	Extrinsic (X)		Intrinsic (I)		$\Delta$(X–I)	
	PS	LS	PS	LS	PS	LS
	I	II	III	IV	V	VI
*Before ownership*						
4–3 years hence	− 0.002	− 0.090	0.189**	0.024	− 0.191	− 0.115
	(0.08)	(0.08)	(0.08)	(0.10)	(0.12)	(0.12)
3–2 years hence	0.140	0.065	0.101	0.161*	0.039	− 0.095
	(0.09)	(0.08)	(0.09)	(0.09)	(0.13)	(0.12)
2–1 years hence	0.218***	0.077	0.221**	0.170*	− 0.003	− 0.093
	(0.08)	(0.08)	(0.09)	(0.09)	(0.12)	(0.12)
12–5 months hence	0.180*	0.160*	0.181**	0.293***	− 0.001	− 0.133
	(0.09)	(0.08)	(0.09)	(0.10)	(0.13)	(0.13)
4–0 months hence	0.522***	0.318***	0.191	0.313**	0.331*	0.005
	(0.12)	(0.10)	(0.14)	(0.12)	(0.19)	(0.16)
*After ownership*						
0–4 months	0.642***	0.351***	0.159	0.392***	0.484***	− 0.040
	(0.12)	(0.11)	(0.13)	(0.13)	(0.17)	(0.17)
5–12 months	0.384***	0.237***	0.202**	0.145	0.182	0.091
	(0.10)	(0.09)	(0.10)	(0.11)	(0.14)	(0.14)
1–2 years	0.135	0.092	0.151*	0.277***	− 0.016	− 0.185
	(0.09)	(0.08)	(0.09)	(0.09)	(0.13)	(0.12)
2–3 years	0.145	0.092	0.143	0.236**	0.002	− 0.145
	(0.11)	(0.10)	(0.11)	(0.10)	(0.15)	(0.14)
3–4 years	0.104	0.073	0.215**	0.323***	− 0.110	− 0.250*
	(0.10)	(0.09)	(0.09)	(0.10)	(0.14)	(0.13)
4–5 years	0.121	0.031	0.112	0.237**	0.009	− 0.206
	(0.11)	(0.10)	(0.13)	(0.12)	(0.17)	(0.15)
5–6 years	0.225*	0.089	0.218*	0.204*	0.007	− 0.115
	(0.12)	(0.10)	(0.12)	(0.11)	(0.17)	(0.15)
6 or more years	0.307***	0.152*	0.323***	0.350***	− 0.016	− 0.198
	(0.11)	(0.09)	(0.11)	(0.10)	(0.15)	(0.14)
*Differences*						
PS(4–0 months)–LS(4–5 years)	0.491***		− 0.046		0.537**	
	(0.14)		(0.17)		(0.22)	
PS(0–4 months)–LS(5–6 years)	0.554***		− 0.045		0.599***	
	(0.14)		(0.15)		(0.21)	
PS(5–12 months)–LS(5–6years)	0.295**		− 0.002		0.297*	
	(0.12)		(0.12)		(0.17)	
Individual controls	Yes	Yes	Yes	Yes	Yes	Yes
Age fixed effects (FE)	Yes	Yes	Yes	Yes	Yes	Yes
Time and region FE	Yes	Yes	Yes	Yes	Yes	Yes
Individual FE	Yes	Yes	Yes	Yes	Yes	Yes
No. of observations	71,779	71,779	46,486	46,486	118,265	118,265
No. of individuals	11,871	11,871	7,681	7,681	19,552	19,552
No. of home owners	399	399	282	282	681	681
R$^2$	0.05	0.05	0.05	0.04	0.05	0.05

Standard errors in parentheses. $\Delta$(X–I) indicates the specifications that show the difference in the coefficients between extrinsically and intrinsically oriented individuals. Specifications V and VI show the difference between specifications I and III and between II and IV, respectively. These differences are estimated by including the interaction terms of all covariates with the dummy equal to one for the extrinsically oriented individuals in specifications V and VI (full interaction model)

Significance levels: *$$.05<p<.1$$, **$$.01<p<.05$$, ***$$p<.01$$

**Fig. 4:**
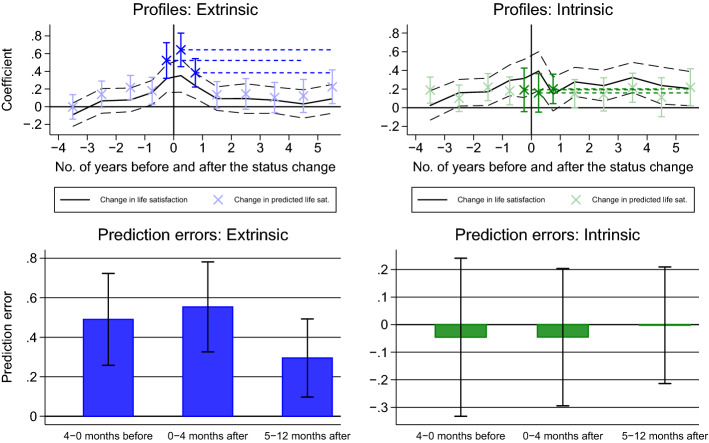
Graphical representation of the estimated patterns in actual and predicted life satisfaction and of the prediction errors around the status change to home owner for extrinsically and intrinsically oriented home buyers. *Note*: 90% confidence intervals are indicated. For a description, see the notes to Fig. 1 *Source*: SOEP

**Fig. 5:**
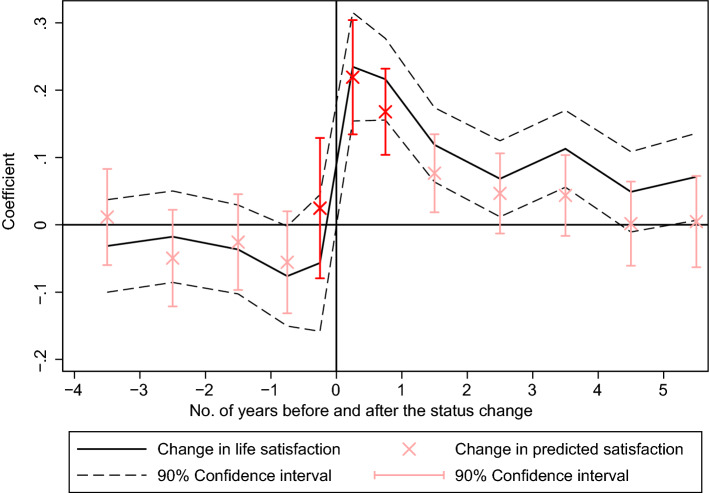
Estimated patterns in actual and predicted life satisfaction around rental relocation. *Note*: This figure is based on the estimated coefficients in Table 6. The black solid line shows the coefficients of specification II and the red x-marks indicate the coefficients of specification I *Source*: SOEP

Table 2 provides the results from the independent estimation of the profiles for the two groups. Columns I and II provide the estimates for individuals with a predominantly extrinsic value orientation, and columns III and IV provide those for individuals classified as predominantly intrinsically oriented. For ease of presentation, we plot the coefficients and the calculated prediction errors in Fig. 4. The coefficients reveal a rather distinct pattern. While the individuals who are classified as extrinsically oriented make systematic prediction errors shortly before and after their status change, the intrinsically oriented individuals do not. With errors of 0.491 and 0.554 points for extrinsically oriented home buyers shortly before and after the status change, respectively, the magnitude of the errors is almost double the size of the errors we calculate in Table 1 for the full sample.^17^

Columns V and VI show the differences across the two groups in the predicted life satisfaction profile and the realized life satisfaction profile. We estimate these differences by including the interaction terms of all covariates with a dummy equal to one for the extrinsically oriented individuals. The results show that the profiles of the two groups differ particularly with regard to the predicted life satisfaction shortly before and after the home purchase. Whether the errors differ systematically between the two groups, as Hypothesis 2 suggests, is statistically addressed at the bottom of columns V and VI. The empirical tests show that the differences for the prediction errors in all three studied time periods are statistically significantly larger for the extrinsically than for the intrinsically oriented home buyers.^18^

In sum, the results provide first evidence that extrinsically oriented home buyers, on average, make larger prediction errors about their future satisfaction with life around the purchase of property than intrinsically oriented home buyers. This indicates that an extrinsic belief system might serve as a sub-optimal heuristic when facing the decision of buying a home, as it is related to an overestimation of the benefits of home ownership. Moreover, it provides evidence for the relevance of differences in underlying beliefs about preferences as a driver of the prediction error.

## Conclusions

This study explores whether home owners systematically overestimate the well-being derived from living in a privately owned house. For this, we jointly analyze people’s expectations regarding their future satisfaction and their actually experienced satisfaction with life later on. This allows us to study whether home buyers, on average, hold accurate beliefs—a cornerstone of standard economics—when facing the house purchase.

The results offer evidence in line with our hypothesis that home buyers systematically overestimate their future life satisfaction just before as well as just after having relocated to their acquired dwelling. This provides support for the speculation that home buyers potentially rely on biased beliefs regarding the long-term benefits of home ownership in the decision-making process, at least if they consider the purchase of property as a main reason for moving. The finding backs the general notion that people overestimate the satisfaction consequences of certain life achievements. From this observation, it is, however, difficult to assess whether the prediction errors are primarily driven by biased beliefs people hold, for example, about their individual preferences. We therefore investigate the heterogeneity in prediction errors across groups with different life goals, reflecting differences in underlying beliefs about preferences or, more generally, what goals should be pursued in life to satisfy needs. Specifically, we study differences regarding the preferability of extrinsic versus intrinsic life goals. We find that home buyers with extrinsically oriented life goals compared to those with intrinsically oriented ones tend to make bigger prediction errors. This result provides evidence for biased beliefs and demonstrates the crucial role of the heterogeneity in people’s beliefs regarding the well-being consequences of certain decisions.

Our study questions the ancillary role that is ascribed to beliefs in most economic applications. If people predict the utility from decision outcomes based on beliefs about their preferences, individuals’ choices would not reveal true preferences, but rather beliefs regarding preferences. Our findings provide evidence in this direction by showing that the accuracy of people’s predictions depends on their belief system. A further investigation of the role of beliefs is a promising topic for future research, as it affects fundamental theoretical assumptions of the economic approach. For example, one could study to what extent beliefs about the utility derived from goods or experiences are influenced by factors such as culture and formal institutions, advertising or, on the individual level, parenting and education. Such endogeneity of people’s beliefs complements what has up to now been discussed under the notion of endogenous preferences in economics [see, e.g., (Bowles, 1998)].

Another perspective on the role of beliefs in economics is that the formation of beliefs plays a fundamental role in the process when people are trying to achieve short- and long-term goals in life. In this process, accuracy might not be the only objective, as beliefs also serve the important purpose of motivating people so that they persevere in applying effort to achieve goals (see (Bénabou & Tirole, 2016) or (Epley & Gilovich, 2016) on motivated beliefs). This instrumental aspect emphasizing the enhancement of self-efficacy is complemented by other motives, as people might want to share beliefs in accordance with their peer group or their self-image. Other reasons for belief distortions are discussed by Brunnermeier and Parker (2005), who argue that a small bias in subjective beliefs can lead to first-order gains due to increased anticipatory utility (see also (Loewenstein & Molnar, 2018) for a review on belief-based utility). Accordingly, people might (implicitly) trade-off belief-based utility in the short-term for accuracy in the long-term. Whether this trade-off is sub-optimal, reducing individuals’ welfare overall, is difficult to judge however, also within our framework. The less people value and consume the dream of home ownership per se beforehand, the more likely will mispredicted utility be related to a welfare loss due to inaccurate beliefs.

From a general perspective, it is crucial that economic analysis gains a better understanding of the role of individuals’ beliefs as a driver of mispredicted utility and potentially sub-optimal behavior. Such a research enterprise also involves the forces and actors that influence people’s (life) goals and thus their beliefs. If these actors pursue private interests, influence might translate into attempts at manipulation. It is thus important that the conditions under which biased beliefs evolve and influence decision-making processes are identified, an account that economics has not offered so far.

## A Appendix

See Tables 3, 4, 5, 6, 7 and 8 and Fig. 5.

**Table 3:** Descriptive statistics for the two sub-samples of always tenants and prospective home owners *Source*: SOEP

	Always		Prospective	
	tenants		home owners	
	Mean	SD	Mean	SD
*Well-being measures*				
Life satisfaction	6.80	1.83	7.25	1.53
Predicted life satisfaction in 5 years	6.93	2.01	7.50	1.65
*Demographics*				
Female	0.53	0.50	0.50	0.50
Age	45.70	16.75	38.96	11.40
No. of years schooling	11.59	2.52	12.70	2.74
German	0.88	0.32	0.93	0.25
*Marital status*				
Single	0.24	0.43	0.20	0.40
Married	0.59	0.49	0.73	0.44
Separated	0.02	0.14	0.01	0.11
Divorced	0.09	0.28	0.05	0.22
Widowed	0.06	0.25	0.01	0.09
*Labour force status*				
Working	0.59	0.49	0.80	0.40
Unemployed	0.07	0.26	0.03	0.18
Not working	0.12	0.32	0.07	0.25
In education	0.02	0.15	0.02	0.14
Maternity leave	0.02	0.13	0.03	0.18
Some work	0.03	0.17	0.02	0.15
Pensioner	0.14	0.35	0.02	0.15
*Household characteristics*				
ln(household net income)	7.53	0.54	7.83	0.45
No. of children in HH	0.58	0.92	0.86	1.02
Size of household	2.69	1.29	3.07	1.22
No. of observations	126,276		8,811	
No. of individuals	25,081		839	

The number of observations and individuals relate to the sample used for the analysis in specifications I and II of Table 1

**Table 4:** Regression of predicted and actual life satisfaction around the status change to home owner: Full estimation output of Table 1

	PS	LS
	I	II
*Before ownership*		
4–3 years hence	0.079	− 0.047
	(0.06)	(0.06)
3–2 years hence	0.144**	0.108*
	(0.06)	(0.06)
2–1 years hence	0.260***	0.171***
	(0.06)	(0.06)
12–5 months hence	0.248***	0.235***
	(0.06)	(0.06)
4–0 months hence	0.359***	0.327***
	(0.08)	(0.07)
*After ownership*		
0–4 months	0.443***	0.415***
	(0.08)	(0.08)
5–12 months	0.342***	0.227***
	(0.07)	(0.07)
1–2 years	0.161**	0.166***
	(0.06)	(0.06)
2–3 years	0.137*	0.165**
	(0.07)	(0.07)
3–4 years	0.192***	0.201***
	(0.07)	(0.06)
4–5 years	0.123	0.105
	(0.08)	(0.07)
5–6 years	0.220***	0.141*
	(0.08)	(0.07)
6 or more years	0.306***	0.179***
	(0.07)	(0.07)
Married	Ref.	Ref.
Single	0.007	− 0.026
	(0.04)	(0.04)
Separated	0.239	− 0.288
	(0.05)	(0.06)
Divorced	0.115**	0.029
	(0.05)	(0.05)
Widowed	0.021	− 0.179***
	(0.07)	(0.07)
Working	Ref.	Ref.
Unemployed	− 0.268***	− 0.638***
	(0.03)	(0.03)
Not working	− 0.081***	− 0.162***
	(0.03)	(0.03)
In education	0.032	− 0.045
	(0.04)	(0.04)
Maternity leave	0.055	0.070*
	(0.04)	(0.04)
Some work	− 0.074**	− 0.202***
	(0.03)	(0.03)
Pensioner	− 0.119**	− 0.210***
	(0.06)	(0.05)
No. of years schooling	0.027***	0.004
	(0.01)	(0.01)
ln (household net income)	0.249***	0.554***
	(0.02)	(0.02)
German	− 0.068	− 0.013
	(0.08)	(0.08)
No. of children in HH	0.018	0.068***
	(0.02)	(0.02)
Size of household	− 0.067***	− 0.138***
	(0.01)	(0.01)
Individual controls	Yes	Yes
Age fixed effects (FE)	Yes	Yes
Time and region FE	Yes	Yes
Individual FE	Yes	Yes
No. of observations	126,276	126,276
No. of individuals	25,081	25,081
R$^2$	0.05	0.04

Standard errors in parentheses. Significance levels: *$.05<p<.1$, **$.01<p<.05$, ***$<.01$

**Table 5:** Regression of predicted (PS) and actual life satisfaction (LS) around the status change to home owner: heterogeneity and robustness *Source*: SOEP

	Sample 1		Sample 2		Sample 3		Sample 4	
	PS	LS	PS	LS	PS	LS	PS	LS
	I	II	III	IV	V	VI	VII	VIII
*Before ownership*								
4–3 years hence	0.078	− 0.033	0.015	− 0.050	− 0.102	− 0.084	0.049	− 0.063
	(0.07)	(0.07)	(0.05)	(0.05)	(0.13)	(0.11)	(0.06)	(0.06)
3–2 years hence	0.131*	0.115*	0.035	0.009	− 0.084	− 0.136	0.129*	0.089
	(0.07)	(0.07)	(0.05)	(0.05)	(0.13)	(0.13)	(0.07)	(0.07)
2–1 years hence	0.254***	0.155**	0.176***	0.116***	0.081	− 0.022	0.272***	0.191***
	(0.07)	(0.07)	(0.05)	(0.05)	(0.13)	(0.13)	(0.06)	(0.07)
1–0 year hence					0.061	0.125		
					(0.13)	(0.13)		
12–5 months hence	0.215***	0.237***	0.186***	0.087*			0.267***	0.238***
	(0.07)	(0.07)	(0.05)	(0.05)			(0.07)	(0.07)
4–0 months hence	0.375***	0.323***	0.199***	0.206***			0.407***	0.362***
	(0.10)	(0.08)	(0.07)	(0.06)			(0.09)	(0.08)
*After ownership*								
0–4 months	0.466***	0.468***	0.319***	0.305***			0.457***	0.429***
	(0.09)	(0.08)	(0.06)	(0.05)			(0.09)	(0.09)
5–12 months	0.362***	0.256***	0.272***	0.224***			0.361***	0.236***
	(0.07)	(0.07)	(0.05)	(0.05)			(0.07)	(0.07)
0–1 year					0.071	0.028		
					(0.13)	(0.13)		
1–2 years	0.169**	0.197***	0.129***	0.152***	− 0.029	− 0.058	0.188**	0.182***
	(0.07)	(0.07)	(0.04)	(0.04)	(0.13)	(0.13)	(0.08)	(0.07)
2–3 years	0.136*	0.170**	0.090*	0.125***	− 0.035	− 0.022	0.147*	0.174**
	(0.08)	(0.08)	(0.05)	(0.05)	(0.14)	(0.14)	(0.09)	(0.08)
3–4 years	0.216***	0.210***	0.136***	0.149***	− 0.049	0.063	0.183**	0.227***
	(0.08)	(0.07)	(0.05)	(0.05)	(0.15)	(0.14)	(0.09)	(0.08)
4–5 years	0.064	0.017	0.147***	0.170***	0.021	0.091	0.186*	0.166*
	(0.09)	(0.08)	(0.05)	(0.05)	(0.16)	(0.14)	(0.11)	(0.09)
5–6 years	0.196**	0.137*	0.134**	0.158***	− 0.003	0.056	0.218**	0.145*
	(0.09)	(0.08)	(0.06)	(0.05)	(0.15)	(0.15)	(0.11)	(0.09)
6 or more years	0.285***	0.170**	0.165***	0.173***	0.016	− 0.016	0.260***	0.164**
	(0.08)	(0.07)	(0.05)	(0.05)	(0.13)	(0.14)	(0.10)	(0.08)
*Differences:*								
PS (4–0 months)–LS (4–5 years)	0.357***		0.029				0.241**	
	(0.11)		(0.08)				(0.11)	
PS(0–4 months)–LS(5–6 years)	0.329***		0.161**				0.312***	
	(0.11)		(0.07)				(0.10)	
PS(5–12 months)–LS(5–6years)	0.225**		0.113*				0.216**	
	(0.09)		(0.06)				(0.09)	
PS(0–1 year)–LS(5–6 years)					0.0152			
					(0.14)			
No. of observations	124,468	124,468	139,209	139,209	99,042	99,042	126,276	126,276
No. of individuals	24,916	24,916	26,214	26,214	21,588	21,588	25,081	25,081
No. of home buyers	611	611	2136	2136	383	383	839	839
R$^2$	0.05	0.05	0.05	0.04	0.05	0.05	0.05	0.05

Standard errors in parentheses. Sample 1 consists only of home owners who acquired a house (and not an apartment). Sample 2 comprises all the individuals for whom we observe a status change from tenant to owner. Sample 3 refers to people who become owner without changing the dwelling in the previous, the current, or the upcoming year. In Sample 4, the main specifications are estimated using entropy balancing to make tenants more comparable to home buyers. Significance levels: *$.05<p<.1$, **$.01<p<.05$, ***$p<.01$. Significance levels of the prediction errors derived from a z-test

**Table 6:** Regression of predicted (PS) and actual life satisfaction (LS) around rental relocation *Source*: SOEP

	PS	LS
	I	II
*Before relocation*		
4–3 years hence	0.012	− 0.031
	(0.04)	(0.04)
3–2 years hence	− 0.049	− 0.018
	(0.04)	(0.04)
2–1 years hence	− 0.026	− 0.037
	(0.04)	(0.04)
12–5 months hence	− 0.056	− 0.076*
	(0.05)	(0.05)
4–0 months hence	0.025	− 0.057
	(0.06)	(0.06)
*After relocation*		
0–4 months	0.219***	0.235***
	(0.05)	(0.05)
5–12 months	0.168***	0.216***
	(0.04)	(0.04)
1–2 years	0.077**	0.119***
	(0.04)	(0.03)
2–3 years	0.047	0.068**
	(0.04)	(0.03)
3–4 years	0.044	0.113***
	(0.04)	(0.03)
4–5 years	0.002	0.049
	(0.04)	(0.04)
5–6 years	0.005	0.071*
	(0.04)	(0.04)
6 or more years	0.015	0.110***
	(0.04)	(0.04)
*Differences*:		
PS(4–0 months)–LS(4–5 years)	− 0.024	
	(0.067)	
PS(0–4 months)–LS(5–6 years)	0.148***	
	(0.056)	
PS(5–12 months)–LS(5–6years)	0.097**	
	(0.045)	
Individual controls	Yes	Yes
Age fixed effects (FE)	Yes	Yes
Time and region FE	Yes	Yes
Individual FE	Yes	Yes
No. of observations	115,487	115,487
No. of individuals	23,744	23,744
R$^2$	0.05	0.05

Standard errors in parentheses. Significance levels: *$.05<p<.1$, **$.01<p<.05$, ***$p<.01$. Significance levels of the prediction errors derived from a z-test

**Table 7:** Measurement of value orientation

	Survey years	Obs.	Mean	SD
*Items measuring extrinsic orientation*				
Importance of income/earnings	1991, 1994, 1998, 1999	20,309	3.48	0.58
Importance of job success	1991, 1992, 1994, 1995,			
	1998, 1999, 2004, 2008	43,418	2.83	0.93
Importance of fulfilling the own potential	1992, 1995, 2004, 2008	24,785	2.86	0.76
Importance of being able to afford sth.	1992, 1995, 2004, 2008	24,913	3.03	0.60
*Items measuring intrinsic orientation*				
Importance of family	1991, 1994, 1998, 1999	20,294	3.76	0.50
Importance of friends	1991, 1994, 1998, 1999	20,275	3.18	0.68
Importance of being together with friends often	1992, 1995	10,172	2.91	0.70
Importance of leading a happy partnership	1992, 1995, 2004, 2008	24,753	3.56	0.74
Importance of being there for others	1992, 1995, 2004, 2008	24,886	3.17	0.59
Importance of political/social participation	1992, 1995, 2004, 2008	24,804	1.87	0.74

Across surveys, there is a slight variation in the wording of the questions asked. In the years 1991, 1994, 1998, and 1999, people are asked about the importance of the items for their satisfaction, and in the years 1992, 1995, 2004, and 2008 they are asked about their general importance in life today. In the year 1991, the questions only applied to people belonging to an extra East-German sample. There are other items in the questionnaire which we do not use; i.e., importance of work, political influence, domicile, spare time, health, religion, neighbourhood, mobility, a house, environmental protection, having children, and travel

**Table 8:** Descriptive statistics for the samples of extrinsically and intrinsically oriented individuals

	Extrinsic		Intrinsic	
	Mean	SD	Mean	SD
*Well-being measures*				
Life satisfaction	6.71	1.85	6.90	1.77
Predicted life satisfaction in 5 years	6.87	2.03	6.94	1.96
*Demographics*				
Female	0.48	0.50	0.60	0.49
Age	44.04	16.00	49.05	17.40
No. of years schooling	11.49	2.37	11.76	2.74
German	0.89	0.31	0.89	0.32
*Marital status*				
Single	0.27	0.44	0.18	0.39
Married	0.55	0.50	0.65	0.48
Separated	0.02	0.14	0.02	0.13
Divorced	0.10	0.30	0.07	0.26
Widowed	0.06	0.23	0.08	0.27
*Labour force status*				
Working	0.64	0.48	0.51	0.50
Unemployed	0.09	0.28	0.06	0.23
Not working	0.10	0.30	0.15	0.36
In education	0.03	0.16	0.02	0.14
Maternity leave	0.02	0.13	0.02	0.14
Some work	0.03	0.17	0.03	0.17
Pensioner	0.11	0.31	0.21	0.41
*Household characteristics*				
ln(household net income)	7.51	0.54	7.54	0.53
No. of children in HH	0.56	0.90	0.58	0.94
Size of household	2.67	1.27	2.69	1.29
No. of observations	71,779		46,486	
No. of individuals	11,871		7,681	
No. of home owners	399		282	

## Footnotes

1 Reviews are provided by Frey and Stutzer (2014), Kahneman (2003), Kahneman and Thaler (2006) and Morewedge and Hershfield (2015).

2 One exception refers to the accumulating work on the underestimation of adaptation (see, e.g., (Powdthavee & Stutzer, 2014) for a short review or (Odermatt & Stutzer, 2019) for recent evidence).

3 Simonsohn and Loewenstein (2006) support the argument that people’s uncertainty about their own preferences is relevant in the housing market. They argue that their finding of a *contrast effect* exemplifies that people draw upon a wide range of cues to help them resolve this uncertainty. They show that households which move away from expensive cities to less expensive ones spend more in their destination city, because their previous exposure to high prices makes prices in the new city seem cheaper. The authors see this as evidence that movers experience some uncertainty about how much they want to spend on housing. Alternative explanations, such as imperfect information, are ruled out.

4 In their study, Sheldon et al. (2010) randomly assigned participants to pursue either extrinsic goals or intrinsic goals for a 4-week period (e.g., either to earn some extra money or to promote social interaction). They show that extrinsically oriented people, who assign a relatively high value to money, success and image, overestimate the emotional benefits of achieving extrinsic goals. The group of more intrinsically oriented individuals were not prone to such misprediction.

5 After 2013, the item about satisfaction with life in 5 years is only asked again in 2018. To avoid a long break in the panel, we only consider data until 2013.

6 A discussion of the separate effects of home ownership and moving on life satisfaction are offered in Diaz-Serrano (2009).

7 A comparison of the mean values of the characteristics shows that home owners differ with regard to some characteristics: compared to tenants, home owners have, on average, a higher level of current and predicted life satisfaction. They also differ in regard to socio-demographic characteristics, as they tend to be younger, have more children and earn more, as well as being more likely to be married and currently employed.

8 To obtain the covariance of the two models, we apply the stacking method described in Weesie (1999).

9 This might not be the case more months before the status change; the period of giving notice required by German law is three months.

10 A full estimation output that includes the control variables for the specifications I and II is presented in Table 4. When comparing the coefficients of the control variables across the two estimates, one can see that for most variables the estimated effects are stronger for current life satisfaction than for predicted life satisfaction. This is very much in line with an earlier finding reported in Odermatt and Stutzer (2019) that people to some extent (but not fully) anticipate the adaptation to life events.

11 Note that we observe statistically significant increases in life satisfaction within the 4 months prior and after the purchase when compared against the coefficient of 2–1 years before the purchase (instead of the comparison against the reference period).

12 In a supplementary analysis, we checked the accuracy of the predictions made at years 1–2 and 2–3 after the home purchase. Note that based on our estimates presented in Fig. 1, the prediction errors two or more years after the status change cannot be assessed, as we need to include further lags in the regressions to calculate the corresponding prediction errors (so far, these observations are grouped in the category “6 or more years” after ownership). Including additional lags for “6–7 years” and “7–8 years” after ownership in addition to a new residual category “8 or more years”, allows us to estimate the difference PS (1–2 years)–LS (6–7 years) and PS (2–3 years)–LS (7–8 years) after ownership. Both estimated differences are small and far from being statistically significantly different from zero (i.e., 0.053 with a *p*-value of 0.58, and 0.046 with a *p*-value of 0.64). We consider this as additional supporting evidence for the relevance of biased beliefs around the move to a newly purchased home.

13 In a supplementary analysis, we look specifically at those people who indicate to have moved because of reasons related to the house or the location they lived in (i.e., size of the dwelling being too small, rental costs, furniture, location, or surrounding of old dwelling). For this sample, we find considerably bigger prediction errors, particularly in the month prior to the move. One interpretation is that people foresee a relieve in the mentioned aspects, but, over time, adapt to these improvements.

14 Note that in this sample of non-movers, we do not have the moving date to construct the leads and the lags. We therefore rely on the information on the status change from one survey to the next to identify the year of the status change.

15 We condition on all the control variables shown in Table 3.

16 The items are measured intermittently and with slight variation in the wording. In the years 1991, 1994, 1998 and 1999, people were asked about the importance of the respective items for their satisfaction, and in the years 1992, 1995 and 2004 they were asked about the general importance of the respective items in their life today. In the year 1991, the question only applied to people belonging to an extra East-German sample.

17 In a robustness check, we conducted the analysis excluding pensioners. The estimates yield similar results. We further checked the sensitivity of the results with respect to the classification of the groups. For this, we simply classified individuals as extrinsic when they indicated income as being very important. Again, the results were similar. The corresponding estimation outputs are available upon request.

18 To assess the significance of the differences in the errors across the two samples, we apply a z-test, a standard procedure to test the equality of regression coefficients across estimations that are based on independent samples [see e.g., (Paternoster et al., 1998)].

## Declarations

### Data Transparency

Our analysis is based on proprietary data collected by the German Institute for Economic Research in Berlin and can thus not be made publicly available. However, the specific data set, i.e., the German Socio-Economic Panel, is publicly available via data agreement with the German Institute for Economic Research.
